# Adipokine Serum visfatin level in pregnancy induced hypertension and uncomplicated pregnancy

**DOI:** 10.12669/pjms.326.10917

**Published:** 2016

**Authors:** Asmat Shaheen, Rubina Nazli, Sadia Fatima, Roshan Ali, Ihsanullah Khan, Salim Khattak

**Affiliations:** 1 Dr. Asmat Shaheen, BDS, M.Phil. Assistant Professor of Biochemistry, Institute of Medical Sciences, Khyber Medical University (KMU), Kohat, Pakistan; 2 Dr. Rubina Nazli, MBBS, PhD. Professor of Biochemistry, Institute of Basic Medical Sciences (IBMS), Khyber Medical University, Peshawar, Pakistan; 3 Dr. Sadia Fatima, MBBS, PhD. Assistant Professor of Biochemistry, Institute of Basic Medical Sciences (IBMS), Khyber Medical University, Peshawar, Pakistan; 4 Dr. Roshan Ali, PhD. Assistant Professor of Biochemistry, Institute of Basic Medical Sciences (IBMS), Khyber Medical University, Peshawar, Pakistan; 5 Ihsanuullah Khan, M.Phil, Institute of Basic Medical Sciences (IBMS), Khyber Medical University, Peshawar, Pakistan; 6 Dr. Salim Khattak, MBBS, FCPS. Department of Surgery, Institute of Medical Sciences, Khyber Medical University (KMU), Kohat, Pakistan

**Keywords:** Visfatin, Pre-eclampsia, Eclampsia, Pregnancy, Hypertension, Normotensive pregnant women

## Abstract

**Background and Objectives:**

Hypertensive disorder in pregnancy is the significant disease that badly affects the maternal and fetal prognosis and lead to higher mortality and morbidity in the prenatal period. Visfatin, potentially a new adipokine has emerged having high contribution in pathogenesis of pre-eclampsia. The objective of the study was to find the level of Visfatin in pregnancy induced hypertension and normal pregnant women.

**Methods:**

This study was carried out in tertiary care hospitals, Peshawar from March-October 2014. A total of 234 pregnant women (gestational age >20 weeks) were included in the study with distribution as Preeclampsia (PE=86), Eclampsia (E=74) and control (N=74). Blood was taken for measuring Visfatin level by Enzyme Linked Immunosorbent Assay (ELISA) technique. SPSS version 19 was used for statistical analysis. Student’s t test was performed to evaluate the mean differences in patients and control.

**Results:**

Serum level of visfatin was significantly higher in pregnancy induced hypertension when compared with control (P value<0.001).: Comparisons of mean value of visfatin with age group of 21-40 years, body mass index (BMI), primary parous and parity 2-4, gestational age of >36 weeks and both systolic and diastolic blood pressure were highly significant in pregnancy induced hypertension when compared with control (p value<0.001).

**Conclusion:**

Pregnancy induced hypertensive women showed increased level of serum Visfatin than normal pregnant women.

## INTRODUCTION

Pregnancy induced hypertension is defined as an incidence of hypertension after 20 weeks of outset with edema and with or without proteinuria. On repeated readings systolic blood pressure is > 140 mmHg and diastolic blood pressure is > 90 mmHg.^1^

Hypertension is complicating at an average 2-3 percent of pregnant women in the world. Various research findings unanimously confirm that maternal mortality rate (MMR) is 400 maternal deaths out of 100,000 live births.^2^ In Africa 1:16 life time risk are recorded which is the highest, compared to the Western nations (1:2800). Eclampsia accounts for 12 percent of such deaths.^3^

The incidence of preeclampsia worldwide is approximately 6-8%. In the United States, the estimated range is 2-6% in healthy nulliparous woman. In developing countries it is a common problem and its frequency varies usually from 1 in 100 to 1 in 1700.^4^ In developing countries, the incidences ranges 4-18% and being considered the 2nd broad obstetric cause of still birth and early neonatal death.^5^ Maternal death from preeclampsia in Pakistan is reported to be 9 to 16.9 percent.^6^ The incidence of preeclampsia varies across the world and it can be due to geographical, social, economic and racial differences.^7^

The etiology of preeclampsia is unidentified but it is commonly inferred that pathogenesis of PE is catalysed by adipokines.^2^ Human nicotinamide phosphoribosyltransferase (Nampt) is an enzyme that is determined by pre-b cell colony enhancing factor-1 (PBEF1) gene. It is also called visfatin which is known as pre-B cell colony attractive factor1. Nampt is adipokines and is restricted to the blood stream and has a variety of functions including the enhancement of B cell maturation and inhibits neutrophils apoptosis. It also activates insulin receptors and has insulin mimetic effects.^8^,^9^ Several groups have reported that Nampt/PBEF protein is a dimeric phosphoribosyl transferase enzyme concerned in nicotinamide adenine dinucleotide (NAD) biosynthesis. Nampt/PBEF was re-identified as a new visceral fat derived hormone named Nampt. The important role of adipokines has been confirmed recently in normal human gestation and in pregnancy complications (World Health Organization 2003). Increased circulating maternal concentration of resistin, leptin and visfatin^10^,^11^ were reported in gestational diabetes pregnant women. Some of these are limited reports to pregnancy complications such as PE.^12^,^13^ Along gestation different studies have reported concentration of maternal visfatin. There is a great requirement to initiate such fundamental works on the concentration of insulin mimetic Nampt during pregnancies with PE.^8^,^14^

The aim of the current study was to investigate the level of visfatin in women with pregnancy induced hypertension and in normotensive pregnant women to know the part of visfatin in the pathophysiology of preeclampsia.

## METHODS

The cross sectional study was approved by ethical board of Khyber Medical University and all the participants provided written informed consent. The participants of this study were 234 pregnant women between the ages of 18-45 years with gestational age of >20 weeks. They were registered from three major hospitals of Peshawar- Khyber Pakhtunkhwa (KPK), Pakistan, i.e., Lady Ready Hospital (LRH), Khyber Teaching Hospital (KTH) and Hayatabad Medical Complex (HMC). One hundred and sixty (160) women with pregnancy induced hypertension were randomly selected. Seventy four (74) healthy pregnant females matched for age, body mass index (BMI) and socioeconomic status were selected as a control group. The main inclusion criteria for the patients of this study was gestational age >20 weeks with persistent high blood pressures (140/90mmHg or more), gross proteinuria (positive on dipstick method) and with or without oedema.

The participants with the present and past history of diabetes mellitus, hypertension, liver diseases, renal diseases, multiple pregnancies and any drug effecting adipokines were excluded from the study. A complete antenatal history of previous pregnancies was also taken by the researcher like age at marriage, prior history of still birth babies, low birth weight, pregnancy complicated by hypertension. Moreover, a complete current antenatal history was also obtained including the history of sign and symptoms of pre-eclampsia (fluid retention, severe headache abdominal pain, low urine output, haematuria, altered reflexes, dizziness, CNS irritability, nausea, excessive vomiting, and quick weight gain).

Height of the participants were measured with vertical calibrated scale. The measurement was recorded in centimeters. Weight of participants was calculated in the standing position using a standard health weighing scale. All the measurements were measured by the main researcher using the same scales for weight and height measurements. From the measurement of weights and heights BMI was calculated by the formula “BMI = Weight in Kg/Height in (meter)”.^15^

Standard mercury Sphygmomanometer was used to measure both systolic and diastolic blood pressure at left arm. Three times blood pressure was recorded and their mean values were taken.^16^ Hypertensive women were screened for proteinurea by using dipstick method. About 5ml of blood was taken under aseptic technique and collected in EDTA and Gel tubes for further process. To get a cell free serum each blood sample was centrifuged for 10 minutes at 3000 rpm. Around 4 ml of uncoagulated blood was collected in vacutainer tube. Serum visfatin levels (ng/ml) were determined by Enzyme linked immune sorbent assay (ELISA). The assay was conducted using Biovision Human visfatin ELISA kit (Biovision Research Products- CA94043. USA). Urine albumin was determined by dipstick method. These investigations were performed in IBMS-KMU Peshawar.

### Statistical Analysis

Using software SPSS version 19 the statistical analysis of the data was performed. The mean of data was presented. To calculate mean differences in cases and normal subjects the Student’s *t* test were used. P < 0.05 was considered as significant. Significance with the means of different groups was expressed in term of ‘P’ value.

## RESULTS

Baseline characteristics of the patients and controls are summarized in Table-I. The mean differences in the systolic blood pressure, diastolic blood pressure and monthly incomes were statistically significant when compared with normotensive pregnant women. The mean age of patients group was 30.8±8.48 years and control was 30.9±7.54 years. Age at marriage in patients and control groups shows no significance. Mean BMI in the patient group was 29.4±5.62 kg/m^2^ and in control was 30.7±6.26 Kg/m^2^ which was statistically insignificant. The mean differences in the monthly incomes were statistically significant when compared between normotensive pregnant women.

**Table-I:** Comparison of socio-demographic characteristics of the study groups.

Characteristics	Patients (n=160)	Control (n=74)	t. test	P. value
Age (years)	30.8 ± 8.48	30.9 ± 7.54	-0.129	0.897
Age at marriage (years)	15.4 ± 2.42	15.5 ± 2.21	-0.226	0.822
Weight (kg)	70.4 ± 10.49	72.3 ± 10.73	-1.301	0.195
Height (m)	1.5 ± 0.11	1.5 ± 0.11	0.717	0.474
BMI (kg/m^2^)	29.4 ± 5.62	30.7 ± 6.26	-1.607	0.109
Systolic BP (mmHg)	159.3 ± 22.16	106.8 ± 11.33**	19.221	0.000
Diastolic BP (mmHg)	106.7 ± 12.91	68.5 ± 9.606**	22.720	0.000
Monthly income (PKR per month)	7578.7 ± 6121.16	11831.0± 5235.72**	-5.164	0.000

** The mean difference is highly significant at the level (p value < 0.001).

**Table-II:** Comparison between socio demographic and clinical characteristics among the study groups.

Age Group (years)	Patients (n=160) N (%)	Control (n=74) N (%)
< 20	21(13.12)	4(5.4)
21-30	61(38.12)	35(47.3)
31-40	54(33.75)	27(36.5)
≥ 40	24(15.0)	8(10.8)
*Gestational age (weeks)*		
27-30	15(9.3)	11 (4.9)
31-35	72 (45.0)	12 (16.2)
>36	73 (45.62)	51 (68.9)
*Parity*		
Primiparous	86 (53.75)	35 (47.2)
2-4	57 (35.62)	38 (51.35)
5-10	15 (9.37)	1 (1.35)
>10	2(1.25)	0
*Urinary Albumin*		
Nil	0	71 (95.9)
+	23 (14.3)	1 (1.14)
++	64 (40)	1 (1.14)
+++	69 (43.12)	1 (1.14)
++++	4 (2.5)	0

**Fig.1:**
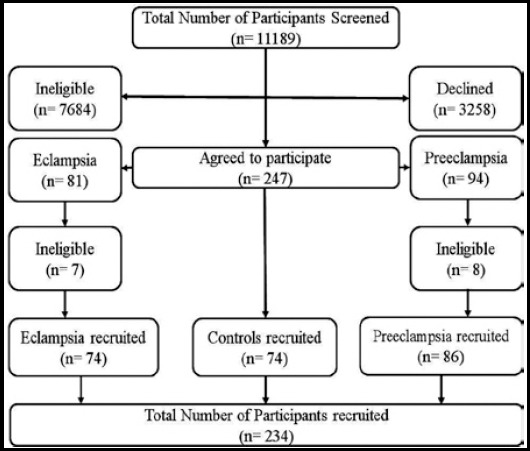
Enrollment and Flow of participants during study.

Majority of the cases were primiparous 80 (53.75%) who belonged to patient group. No urinary protein was present in 71 (95.9%) in control group. 69 (43.12%) patients showing +++ urinary protein when compared with control. The mean differences in visfatin level among the patient and the normotensive pregnant women are shown (Table-III). The mean difference of visfatin in PE and E were highly significant (p<0.001) when compared with control.

**Table-III:** Comparison of mean visfatin levels among the study groups.

Participants	Number	Mean ± SD visfatin (ng/ml)	t. test	P. value
Preeclampsia	86	4.9 ± 3.05	6.773	0.000
Eclampsia	74	3.5 ± 2.05	4.529	0.000
Control	74	2.2 ± 1.57	-	-

**The mean difference is highly significant at the level (p value < 0.001).

Comparisons of mean value of visfatin in different reproductive and clinical parameters are summarised in Table-IV. The mean difference of maternal age group >20 were statistically significant when compared with control. Gestational age in patients and control groups shows significance difference at >36 weeks. The mean differences in the BMI, normal, overweight and obese were statistically significant when compared between normotensive pregnant women. In parity group statistical significance was found in primary parous and parity 2-4. Systolic and diastolic blood pressures were found statistically significant in the patients when compared with control.

**Table-IV:** Comparisons of mean visfatin in different reproductive and clinical parameters.

Parameters	Patient (n=160) (Mean ± SD)	Control (n=74) (Mean ± SD)	t. test	P. value
*Age group (Years)*				
< 20	3.2± 2.50	3.8± 2.35	-.325	0.751
21-30	4.1± 2.64	2.0± 1.35**	4.446	0.000
31-40	4.7± 2.92	2.3± 1.87**	3.870	0.000
41-50	4.2± 2.50	2.1± 1.22*	2.244	0.032
*BMI (Kg/m^2^)*				
Normal	4.4± 2.84	2.5± 1.60*	2.370	0.021
Overweight	4.4± 2.69	2.1± 1.34*	3.673	0.001
Obese	4.1± 2.67	2.1± 1.70**	4.142	0.000
*Gestational age (Weeks)*				
27-30	3.7± 2.37	2.2± 1.46	1.784	0.087
31-35	4.4± 2.84	2.9± 2.1	1.741	0.085
>36	4.2± 2.67	2.0± 1.42**	5.446	0.000
*Parity*				
Primparous	4.0± 2.55	2.1± 1.40**	4.001	0.000
2-4	4.2± 2.75	2.3± 1.70**	3.878	0.000
5-10	5.4± 3.04	0.07 (1)	NC	
>10	8.7± 0.4	(0)	NC	
*Systolic BP (mm/Hg)*				
< 150	4.1± 2.54	2.2± 1.56**	5.496	0.000
> 150	4.4± 2.87	0.56 (one case)	NC	
*Diastolic BP (mm/Hg)*				
< 110	4.2± 2.58	2.2± 1.57**	5.981	0.000
> 110	4.3± 2.90	0	NC	

NC: Not calculated

** The mean difference is highly significant at the level (p value < 0.001).

* The mean difference is significant at the level (p value < 0.01).

## DISCUSSION

Preeclampsia is found as an immediate hypertension threat to life which also promotes the cardio vascular disorder in pregnancy and is associated with increased cardiovascular disease (CVD) develops risk later in life. Besides it is also the prime cause of maternal and fetal mortality and morbidity in pregnancy.^17^ Preeclampsia contributes to cardiovascular risk factors in association with the metabolic syndrome meaning insulin resistance, subclinical inflammation, and obesity. Indeed visfatin/Nampt levels are increased during these pathological conditions, whereas several studies have yet to confirm the cause of preeclampsia due to enhanced level of visfatin/Nampt. Our findings reveal increased serum level of visfatin/Nampt in pregnancy induced hypertension when compared to their matched pregnant controls. Some studies have observed increased maternal serum visfatin levels in preeclamptic patients compared to their matched pregnant controls.^18^ There are other groups which hold different perception and to them decreased expression of visfatin, may contribute to the pathogenesis of preeclampsia. However down regulation of maternal plasma visfatin/Nampt concentration in women with mild preeclampsia and to a higher extent in women with severe preeclampsia was reported by Hu et al.^19^

Fasshauer *et al*. has established positive linkage between visfatin/Nampt serum concentrations and different age groups participating in study.^20^ In our study we also found positive association between serum Visfatin concentrations and different age groups. The age group of >20 years was highly significant in our study.

There is lack of an association between circulating visfatin and BMI.^21^ Some of the studies show positive association between visfatin concentration and BMI.^22^ Negative association have also reported between the two factors.^23^ Different groups have reported, both higher^24^ and lower^25^ concentration of visfatin in obese, than in normal subjects. Our results however do not confirm to these findings and reveal that the association between circulating visfatin and BMI are statistically significant in all normal, overweight and obese subjects.

Fasshauer et al.^20^ and subsequently Adali et al.^26^ investigated maternal concentrations of circulating visfatin were higher in patients with preeclampsia than in normal pregnant women of >27 weeks of gestational age. Our study shows increase serum Visfatin concentrations with gestational age >36 weeks in pregnancy induced hypertension which are statisticaly significant as compared to normal pregnancies. In this context other studies reveal that changes in adipokines are related with adaptations to gestation in addition to complications of pregnancy.^27^

The relationship between visfatin and blood pressure has gained significance for the last few years. Fasshauer et al. has established positive linkage between visfatin serum concentrations and BP in preeclampsia.^20^ In our study we found positive association between visfatin serum concentrations and BP in pregnancy induced hypertension and normal pregnant women.

Filippatos et al.^28^ found that the correlation exists between plasma visfatin concentrations with systolic blood pressure. Subsequently this relation was confirmed by Seo et al.^29^ who established that concentrations of plasma visfatin were positively and separately related with diastolic blood pressure in non diabetic healthy Korean women, but not in men.

The variation in findings could attributes to couple of factors such as severity of disease, metabolic abnormality, the blood sampling time, serum or plasma from blood, of study subjects. It reveals that increased level of visfatin in PE as compared to the control did not establish any correlation to the visfatin level. The increased expression of visfatin suggests its relation to the pathogenesis of PE. Visfatin appearance might be up-regulated in PE than in control group. It indicates the possible significance of visfatin expression in placenta as worked evidence in the prediction of PE, yet more studies are required to be undertaken to explore the mystery of such medical complication during pregnancy.
